# Specification of blood meals ingested by female sand flies caught in Palestinian foci and identification of their concomitant leishmanial infections

**DOI:** 10.1371/journal.pntd.0008748

**Published:** 2020-10-05

**Authors:** Kifaya Azmi, Gabriele Schonian, Ziad Abdeen

**Affiliations:** 1 Biochemistry and Molecular Biology Department-Faculty of Medicine-Al-Quds University, Abu Deis, The West Bank, Palestine; 2 Al-Quds Nutrition and Health Research Institute, Faculty of Medicine, Al-Quds University, Abu-Deis, The West Bank, Palestine; 3 Al-Quds Public Health Society, Jerusalem, Palestine; 4 Charité University Medicine, Berlin, Germany; Faculté de Pharmacie, FRANCE

## Abstract

Since leishmaniases are zoonotic vector-borne diseases transmitted through the bites of infected female sand flies, identification of the sources of imbibed blood meals and the detection and identification of leishmanial DNA in them are important in discerning animal reservoirs, clarifying the epidemiology and facilitating control of local leishmaniases. CDC light traps, aspirators and sticky paper traps were used to collect sand flies in four Palestinian foci of both, CL and VL. Phlebotomine species identification was based on morphological keys. Female specimens were screened to detect and identify leishmanial infections, using kDNA-PCR and ITS1-PCR, and engorged female specimens were analyzed to identify the origin of their blood meals, using an RDB blood meal assay based on the amplification of the cytochrome b gene (*cyt*b) of vertebrate mitochondrial DNA (mtDNA). Twenty sand fly species, 11 of the genus *Phlebotomus* and nine the genus *Sergentomyia*, were identified. The most abundant species was *Ph*. *papatasi* (33.7%), followed by *Ph*. *sergenti* (21%). Among the 691 female sand fly specimens, 18.5% (128/691) were positive for leishmanial DNA, using the kDNA-PCR and 6.4% (44/691) were positive using the ITS1-PCR. DNA from parasites of the genus *Leishmania* was identified in only 1.5% of the infected sand flies. That of *Leishmania tropica* parasites was detected in six female specimens of *Ph*. *sergenti* and that of *L*. *major* parasites in two female specimens of *Ph*. *papatasi*. Interestingly, two engorged females of the species *Se*. (*Neophlebotomus*) sp. were positive for *L*. *tropica* DNA. Ninety engorged female sand flies of *Ph*. *papatasi* and 104 of *Ph*. *sergenti* had fed on a large variety of vertebrate hosts such as humans, hyraxes, rats, cows, goats and birds. Regarding blood-meals showing a mixture from different species of animal host, hyrax and rat blood was revealed in 8/104 (7.7%) females of *Ph*. *sergenti*. Detection of hyrax blood in engorged female sand flies of the species *Ph*. *sergenti* supports the role of hyraxes being a potential reservoir of *L*. *tropica* in Palestinian regions. Rat blood meals might be significant since a few strains *L*. *tropica* and *L*. *infantum* were isolated from rats. Further studies must be undertaken before conclusions could be drawn.

## Author summary

Leishmaniases are vector-borne tropical diseases caused by more than twenty species belonging to the protozoan genus *Leishmania* and transmitted to humans and some other mammalian species by the bites of female phlebotomine sand flies. In the Palestinian Authority, cutaneous leishmaniasis (CL) is the most common leishmaniasis. This study investigated the Palestinian sand fly fauna, their infection rates and feeding preference determined by applying a kDNA-PCR and an ITS1-PCR. Twenty phlebotomine species were shown to be circulating in the study area. The most abundant species was *Ph*. *papatasi* (33.7%), followed by *Ph*. *sergenti* (21%). Infection by parasites of *L*. *tropica* was confirmed in 6.4%, using an ITS1-PCR. Meals of human blood were the most abundant (78.1%) and those of hyrax blood were identified in the cases of six sand flies, five *Ph*. *sergenti* and one *Ph*. *papatasi*

## Introduction

In the Palestinian Authority, zoonotic cutaneous leishmaniasis (CL) caused mainly by *L*. *tropica* and *L*. *major* is considered a public health problem. That caused by *L*. *major* is a zoonotic disease with the species *Psammomys obesus* and *Phleobotomus papatasi* serving as the reservoir and the vector, respectively. The transmission cycle of CL caused by *L*. *tropica* has not been fully elucidated and relatively little information is available on its epidemiology in the Palestinian Authority. Vectorial competence of *Ph*. *sergenti* has been studied and compared with the three Old World species of *Leishmania L*. *tropica*, *L*. *major* and *L*. *infantum* [1]. *Ph*. *sergenti* showed high specificity for strains of *L*. *tropica* and lost artificially fed infections of *L*. *major* and *L*. *infantum* after voiding the remnants of the digested blood meals. This and similar findings in neighbouring regions suggest that *Ph*. s*ergenti* is, probably, the vector of *L*. *tropica* in the Palestinian Authority [2]. However, none of the sand flies of the species *Ph*. *sergenti* or any other species of the subgenus *Paraphlebotomus*, have been found infected with leishmanial promastigotes in the Palestinian Authority to date. Identification of blood meals ingested by hematophagous arthropods is important for determining their preferred reservoir hosts and, hence, their vectorial capacity for pathogens [3]. Most species of *Leishmania* are zoonotic pathogens. Knowledge of the sand flies and their feeding habits is crucial for incriminating putative reservoir hosts and vectors, and developing effective control strategies [4]. Several molecular biological methods have been applied successfully to blood meal analysis of different haematophagous insects in elucidating their natural host-feeding preferences and host-feeding arrays [5–7].

In this study, molecular biological techniques and tools were applied, i.e., a kDNA PCR and a ITS1-PCR-RFLP to detect and identify species of *Leishmania* within naturally infected sand flies collected in four Palestinian regions. Blood meals from engorged sand flies were analyzed to reveal the animal reservoirs of *L*. *tropica*, using a Reverse Dot Blot PCR (RDB_PCR) technique based on the amplification of the cytochrome b gene (*cyt*b) of vertebrate mitochondrial DNA (mtDNA).

## Materials and methods

### Study area and sample collection

**Fig 1:**
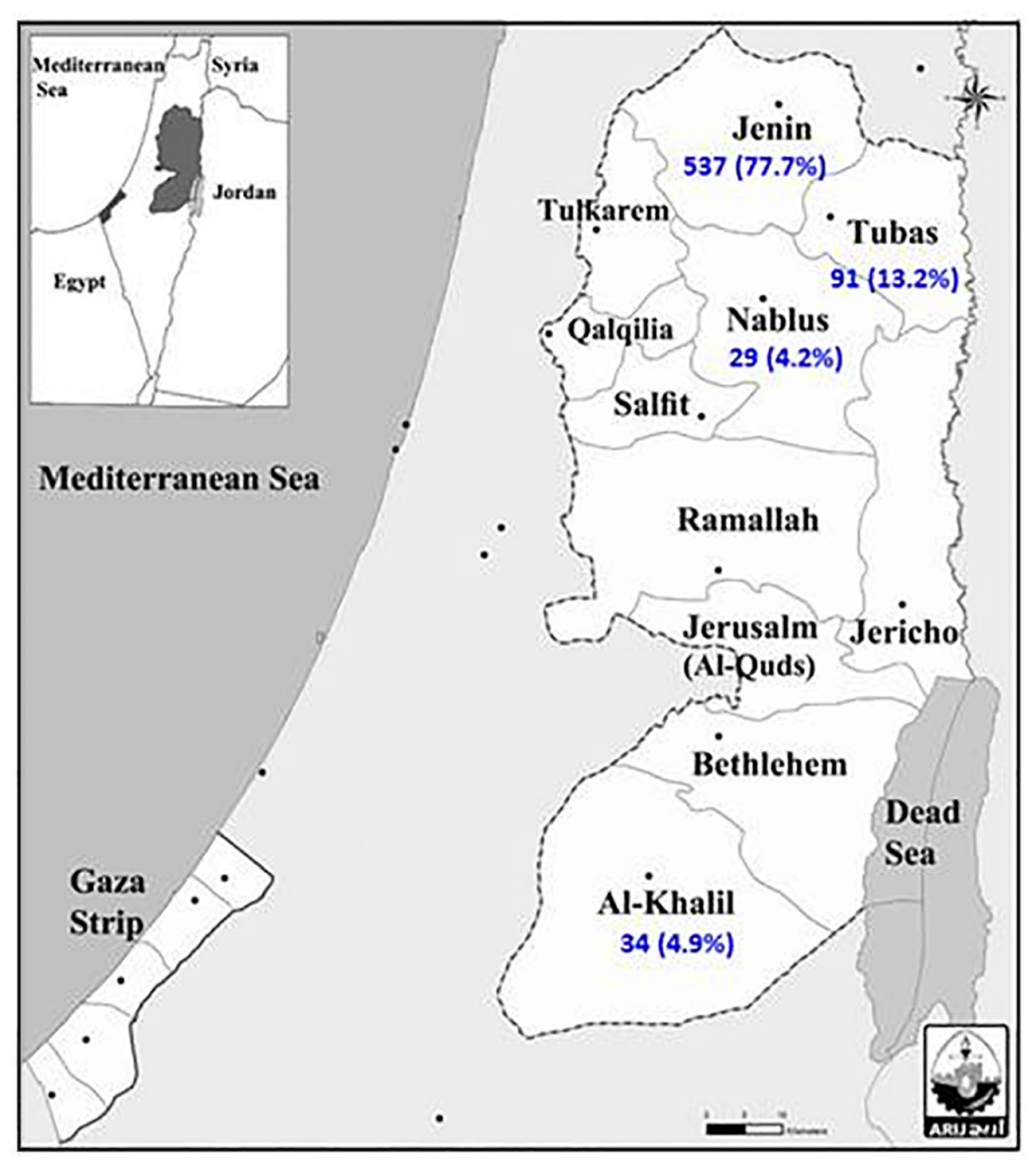
Map of the Palestinian districts investigated, giving the number and the percentages of sand flies collected in each district and in which of the sand flies blood meals were detected and from which animals the blood meals came, including the ones from humans, indicating if leishmanial DNA was detected. Based on the Map of the West Bank and Gaza Strip; Applied Research Institute-Jerusalem (ARIJ), generated by Issa Zboun; using ‘ArcGIS-ArcMap 10.5’. 2018.

Sand flies were collected from May 2010 to November 2012 during their periods of peak activity in four different Palestinian regions: the Jenin, Tubas, Nablus Districts located in the northern part of the West Bank and the Hebron District located in the southern part 30 km south of Jerusalem (Fig 1 map). The Jenin District (32° 20’ N, 35° 8’ E), which is bordered by the Nablus District, is a hilly region of approximately 592 km^2^ with elevations of 90–750 m a.s.l and a mean annual rainfall of 528 mm. The mean annual relative humidity ranges between a mean of 39% in summer and of 84% in winter. The Nablus District (32°13′13″N 35°16′44″E)) has elevations of up to 550 meters a.s.l and annual precipitation rates of approximately 656 mm. The Tubas District (32°19′20″N 35°22′07″E)) with an elevation of 362 meters a.s.l, has a moderate climate with hot and dry summers and cold and wet winters. The average annual temperature is 21°C, and the average annual humidity is 56%. The Hebron District (31°32′00″N 35°05′42″E)) is 930 meters a.s.l., has an average annual temperature of 15°C and an annual level of humidity of 70%. The Ethics Research Committee of the University of Al-Quds (The Palestinian Authority) approved all the activities undertaken. Written informed consent was obtained from residents to have their residences used in the study.

Sand flies were caught using CDC light traps (John W. Hock Company, Gainesville, Florida, USA), by sticky traps made of castor oil–soaked sheets of A4 sized papers (21x29.5 cm) stapled vertically onto wooden stakes at a height of 20 cm [8] and by collecting resting sand flies with an aspirator. Collecting by all three methods was done inside in domestic animal shelters and inside caves and outside near their entrances. Traps were set at fixed sites in the afternoon just before sunset and collected next morning just after sunrise. The collected sand flies were transferred to the lab, washed in a 2% detergent solution and stored in 70% alcohol. The head and either the male genitalia or the female abdominal posterior extremity were mounted on microscope slides in Berlese fluid for morphological identification [9], sexing, and taxonomic identification according to available keys [10–16].

The rest of the bodies of engorged and unfed females were stored on filter paper (GB002; Schleicher and Schuell, Dassel, Germany) at -20°C until used. Genomic DNA was extracted from the abdomen of individual female sand flies), using the high pure PCR template purification kit (Roche Diagnostics GmbH, Mannheim, Germany).

### Detection and identification of *Leishmania* species

Two PCR assays were employed in this study: (1) A multiplex PCR assay to amplify the *Leishmania* conserved region of minicircle kDNA of 120 bp, performed as described by Rodgers et al. [17] that was combined with the amplification of the sand fly house-keeping 12S ribosomal RNA gene, using the modified primers FSF165: 5’TGGCGGTATTTTAGTCTATTCAG and RSF165: 5’AACTGCACCTTGATTTGACAT [18]. The amplification of the sand fly gene was used to rule out false negative results. The 12S rRNA PCR yielded a product of approximately 165 bp for all the phlebotomine sand flies examined. (2) The ITS1 PCR-RFLP was performed according to Schonian et al. [19], using the primer pair L5.8S (5’-TGATACCACTTATCGCACTT-3’) and LITSR (5’-CTGGATCATTTTCCGATG-3’). All PCRs were carried out in 25-μl volume, using the ready PCR mix supreme (Syntezza Bioscience, Jerusalem). Amplicons were analyzed in a 2% agarose gel by electrophoresis at 120V in 1X Tris-Acetate-EDTA buffer (0.04 mM Tris-acetate and 1mM EDTA, pH 8.0) and visualized by UV light after being stained with ethidium bromide (0.3ug/ml). PCRs was considered positive when products of about 120 bp for kDNA and of 165 bp for the 12S r RNA PCRs were observed in the multiplex PCR, and products of about 300 bp were yielded in the ITS1 PCR.

### RFLP analysis of amplified ITS1

DNA control samples of different species of *Leishmania* (*L*. *infantum* MHOM/TN/1980/IPT1, *L*. *tropica* MHOM/IL/1998/LRC-L747 and *L*. *major* MHOM/SU/1973/5ASKH) were obtained from the WHO Reference Center, Hebrew University, Jerusalem. Reaction buffers without leishmanial DNA were included as negative controls. The PCR products (15–20 μl) were digested with the restriction endonuclease *Hae III* or its prototype BsuRI (MBI Fermentas) according to the manufacturer’s instructions. The restriction fragments were subjected to electrophoresis in 2.5% agarose gel at 120V in 1X Tris-Acetate-EDTA buffer. The fragments were visualized by UV light and the size of restriction products was determined.

### Blood meal identification

The PCR assay for revealing the sources of blood meals was based on the amplification of a 344 bp fragment of the conserved region of host mtDNA *cyt*b genes. Amplification and hybridization of host-specific probes to the PCR products was done as proposed by Abbasi et al. [5], with the modification of using dots blots instead of line blots. This combination identified blood meals up to 96 hours after ingestion containing minimal amounts of DNA (>0.1 pg) [5]. Eleven hybridization probes, corresponding to eleven animal species which are possible reservoirs for *L*. *tropica* in the Palestinian Authority, were used: human (*Homo sapiens*), donkey (*Equus asinus*), goat (*Capra hircus*), sheep (*Ovis aries*), cow (*Bos taurus*), canid species, domestic cat (*Felis domesticus*), rock hyrax (*Procavia capensis*), porcupine (*Hystrix indica*), brown rat (*Rattus rattus*) and a general avian probe 1.

## Results

### Phlebotomine sand fly fauna

Of the 757 sand flies collected, 691 were females and 66 were males, all of which were identified to species level. The distribution of the female sand flies by district was: 537 from Jenin, 91 from Tubas, 34 from Hebron and 29 from Nablus, of which 558 (80.8%) and 133 (19.2%) belonged to the genera *Phlebotomus* (*Ph*.) and *Sergentomyia* (*Se*.), respectively (Table 1). The genus *Phlebotomus* was represented by 11 of its subspecies, and their percentages were: *Ph*. *papatasi*, 33.7%; *Ph*. *sergenti*, 21.0%; *Ph*. *tobbi*, 12.4%; *Ph*. *major syriacus*, 4.6*%; Ph*. *perfiliewi transcaucasicus*, 3.9%; and *Ph*. *major neglectus*, 1.4%. The remaining 3.8% belonged to the species *Ph*. *alexandri*, *Ph*. *canaaniticus*, *Ph*. *kazeruni*, *Ph*. *saltiae* and *Ph*. *jacusieli* (Table 1).

**Table 1:** The number of sand flies collected, their species diversity, relative abundance, infection rates and positivity rate for the presence of different species of *Leishmania*.

Species	Total Female SF (%)	Sand flies positive by kDNA PCR (%)	Sand flies positive by ITS1 PCR (%)*	Infected sand flies (%)/*Leishmania* species**/location sites
***Ph*. *alexandri***	2 (0.3)	**1 (50)**		
***Ph*. *jacusieli***	1 (0.1)	**0 (0)**		
***Ph*. *kazeruni***	2 (0.3)	**0 (0)**		
***Ph*. *major neglectus***	10 (1.4)	**3 (30)**	**2 (20)**; Blood meal (n = 2: human)	
***Ph*. *major syriacus***	32 (4.6)	**0 (0)**		
***Ph*. *canaaniticus***	18 (2.6)	**1 (5.6)**		
***Ph*. *papatasi***	233 (33.7)	**19 (8.2)**	**13 (5.58)**; Blood meal (n = 1: human & avian; n = 4: human; n = 8: NI)	**2 (0.4%)/ *L*. *major***^a^ Blood meal (n = 2: human)
***Ph*. *perfiliewi transcaucasicus***	27 (3.9)	**5 (18.5)**	**2 (7.4)**; Blood meal (n = 1: human; n = 1: NI)	
***Ph*. *saltiae***	2 (0.3)	**1 (50)**		
***Ph*. *sergenti***	145 (21)	**47 (32.4)**	**19 (13.1)**; Blood meal (n = 11: human; n = 1: goat; n = 1: human &cow; n = 1: human, cow & goat); n = 5: NI)	**6 (4.1%)/ *L*. *tropica***^b^, Blood meal (n = 1: human & cow; n = 2: human; n = 1: avian & cow)
***Ph*. *tobbi***	86 (12.6)	**26 (30.2)**	**3 (3.4)**; Blood meal (n = 2; human; n = 1: NI)	
**Total *Phlebotomus***	**558 (80.8)**	**103 (18.4)**	**39 (6.9)**	
***Se*. *africana***	24 (3.5)	**4 (16.6)**		
***Se*. *antennata***	3 (0.4)	**1 (33)**		
***Se*. *christophersi***	6 (0.9)	**1 (16.6)**		
***Se*. *dentata***	46 (6.7)	**6 (13)**		
***Se*. *fallax***	9 (1.3)	**0 (0)**		
***Se*. *taizi***	2 (0.3)	**1 (50)**		
***Se*. *theodori***	18 (2.6)	**3 (16.7)**	**1 (5.5)**; Blood meal (n = 1: NI)	
***Se*. *tiberiadis***	13 (1.9)	**3 (23)**		
***Se*. *(Neophlebotomus) sp***.	12 (1.7)	**6 (50)**	**4 (33)**; Blood meal (n = 3: human; n = 1: NI)	**2 (0.3%) *L*. *tropica*** ^c^ Blood meal (n = 1: human; n = 1: NI)
**Total *Sergentomyia***	**133 (19.2)**	**25 (18.8)**	**5 (3.8)**	
**Total**	**691 (100)**	**128 (18.5%)**	**44 (6.4%)**	**10**

*: infected sand flies by ITS1 PCR.

**: infected sand flies by ITS1 PCR RFLP.

^a^, Tubas and Jenin;

^b^, Jenin;

^c^, Tubas;

NI, Not Identified.

Nine species of *Sergentomyia* were identified: *Se*. *dentata* (6.7%), *Se*. *africana* (3.5%), *Se*. *theodori* (2.6%), *Se*. *tiberiadis* (1.9%), *Se*. *(Neophlebotomus) sp*. (1.7%). The remaining 2.7% belonged to the species *Se*. *antennata*, *Se*. *christophersi*, *Se*. *fallax fallax* and *Se*. *taizi*.

A total of 240 sand flies were caught inside houses, consisting of 11 species. *Phlebotomus papatasi* was the most prevalent species among them (191/240, 79.6%), followed by *Ph*. *sergenti* (19/240, 7.9%). Of the 114 phlebotomine flies caught outdoors, *Ph*. *tobbi*, *Se*. *africana*, *Ph*. *perfiliewi*, *Ph*. *sergenti*, *Ph*. *major syriacus*, and *Se*. *dentata* were the most common species. Seven species were caught in animal sheds where *Ph*. *papatasi* (23/66, 34.8%) *and Ph*. *tobbi* (24/66, 36.4%) predominated. Among the 269 sand flies collected from caves in the vicinity of houses and near or inside hyrax burrows, *Ph*. *sergenti* (105/269, 39%) was the most abundant species followed by *Ph*. *tobbi* (33/269, 12.3%). Of the 105 specimens of *Ph*. *sergenti*, 69 (65.7%) were collected from the vicinity of houses and 34 (32.4%) in or near hyrax burrows. Of the 28 specimens of *Se*. *dentata*, 19 (67.9%) were caught near hyrax burrows (Table 2).

**Table 2:** Sand flies collected inside, outside animal sheds and the rock crevasses inhabited by hyraxes in the vicinity of houses.

Species	Animal sheds	Hyrax burrows and caves in the vicinity of houses	Indoor	Outdoor	Total
*Ph*. *major syriacus*	3	19	1	9	32
*Ph*. *mascittii canaaniticus*	5	12	1	0	18
*Ph*. *papatasi*	23	12	191	7	233
*Ph*. *perfiliewi transcaucasicus*	0	6	6	15	27
*Ph*. *sergenti*	7	105	19	14	145
*Ph*. *tobbi*	24	33	6	23	86
*Se*. *africana*	2		1	21	24
*Se*. *dentata*	0	28	7	11	46
*Se*. *(Neophlebotomus) sp*.	0	12		0	12
*other Phlebotomus*	1	15	0	1	17
*other Sergentomyia*	1	27	8	15	51
**Total**	**66**	**269**	**240**	**116**	**691**

### Sand fly blood meal identification

Among the 691 female sand flies collected in the field, 396 (57.3%) contained blood meals, the origins of which could be determined for 301 specimens by the *cyt*b PCR followed by an RDB assay (Table 3). The identification of the blood meals did not succeed in 95 cases, possibly, because of the small amount of blood taken by the sand fly. Blood meals from humans were detected in 172 (32%), 59 (64.8%) and 4 (11.8%) female sand flies collected in Jenin, Tubas and Hebron, respectively.

**Table 3:** The animal and human sources of blood meals imbibed by female sand flies determined by *cyt* b PCR analysis.

Blood fed Sand flies	Blood meal sources
*Ph*. *papatasi* (n = 90)	human (77), human and cow (7), human and avian (2), avian and cow (1), human and goat (1), human, avian and cow (1), human, hyrax, cow & avian (1).
*Ph*. *sergenti* (n = 104)	human (72), human and cow (17), human, cow and goat (5), human and hyrax (4), human and rat (2) hyrax and rat (1), human, cow and avian (1), human and cat (1), goat (1).

*Ph*. *tobbi* (n = 31)	human (22), human, cow and goat (5), human and cow (3), human and avian (1).
*Ph*. *major syriacus* (n = 18)	human (16), human and cow (1), human, cow and goat (1).
*Other *Phlebotomus* (n = 26)	human (20), human and cow (3), human, cow and goat (2), human and donkey (1)
**Sergentomyia (n = 32)	human (28), human and cow (3), human, cow and goat (1)
***Total***	**301**

*Other *Phlebotomus* (n = 26): *Ph*. *saltiae* (n = 2), *Ph*. *alexandri* (n = 2), *Ph*. *major neglectus* (n = 7), *Ph*. *canaaniticus* (n = 6), *Ph*. *perfiliewi transcaucasicus* (n = 9).

** *Sergentomyia* (n = 32): *Se*.*(Neophlebotomus)* sp. (n = 8), *Se*. *africana* (n = 3), *Se*. *antennata* (n = 2), *Se*. *christophersi* (n = 1), *Se*. *dentata* (n = 3), *Se*. *theodori* (n = 6), *Se*. *tiberiadis* (= n = 9).

Of the females examined, 78.4% had fed on a single source. Only human blood was detected in 78.1% (235/301) and only goat blood in 0.3% of these sand flies. However, 48/301 (15.9%) of the sand flies had fed on two types of hosts and 17/301 (5.7%) on three and more different animal hosts. Human blood was found in 77 (32.7%) engorged sand flies of the species *Ph*. *papatasi*, followed by those of *Ph*. *sergenti* (72/235, 30.6%), *Ph*. *tobbi* (22/235, 9.4%), and *Ph*. *major syriacus* (16/235, 6.8%). Hyrax blood was found in six sand flies, five of *Ph*. *sergenti* and one of *Ph*. *papatasi*. Five of these came from the Jenin and one from Tubas Districts.

**Fig 2:**
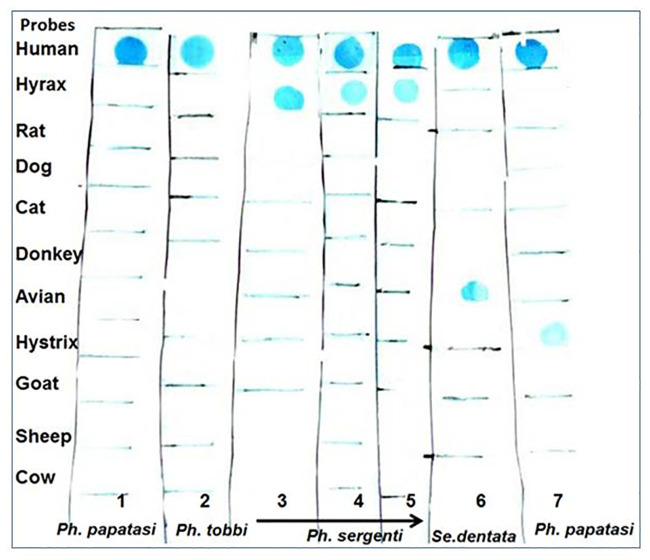
PCR hybridization approach for the identification of the sources of blood imbibed by female sand flies. This is based on the PCR amplification of vertebrate mitochondrial cytochrome b gene sequences combined with reverse dot blot hybridization, which was established and tested according to Boakye et al., 1999, and Abbasi et al., 2009 [3, 5].

Interestingly, the sand flies of the species *Ph*. *sergenti* examined had fed on a variety of vertebrate hosts. Forty-two sand flies had taken a blood meal from a human and a second animal: 34 from cows, four from hyraxes, three from birds, two from rats, one from a cat, one from a donkey and one from a goat. One engorged sand fly had fed on a hyrax and on a rat. Mixed blood meals taken from three different hosts were found in 14 sand flies that had fed on humans, cows and goats, and two others that had fed on humans, cows and birds, and one that had fed on humans, hyraxes, cows and birds (Table 3, Fig 2).

Human blood was detected in 32 (23.3%) of the 133 sand flies of the species S*ergentomyia* tested, four of which had also fed on cow and goat blood.

### Identification of leishmanial parasites from infected sand flies

#### Leishmanial kDNA PCR

**Fig 3:**
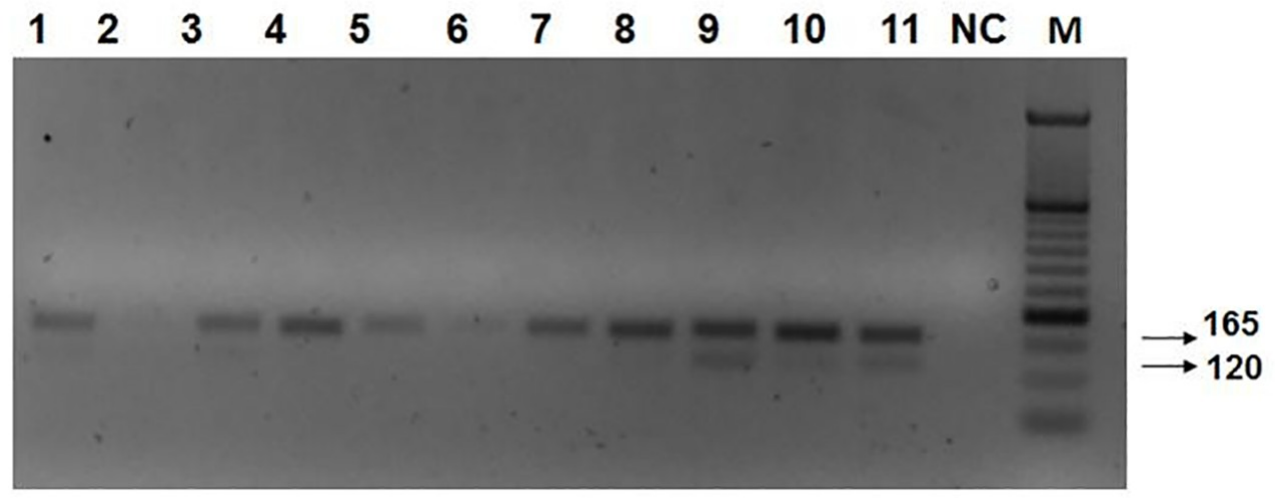
Multiplex PCR amplifying leishmanial kDNA gene sequences (120 bp) and the 12 SL RNA sand fly housekeeping gene (165 bp) in representative sand flies from different districts of the West Bank: NC, PCR negative control; M, molecular weight standard.

Of the 691 sand flies collected, 128 (18.5%) contained leishmanial DNA in them according to the kDNA-PCR method. All positive specimens produced the expected 120 bp-kDNA and the 165 bp sand fly 12S RNA amplification products. PCR inhibition did not occur during screening since all 691 sand fly specimens were positive for the sand fly housekeeping gene 12S SL amplification product (Fig 3, Table 1).

#### *Leishmania* ITS1-PCR-RFLP

**Fig 4:**
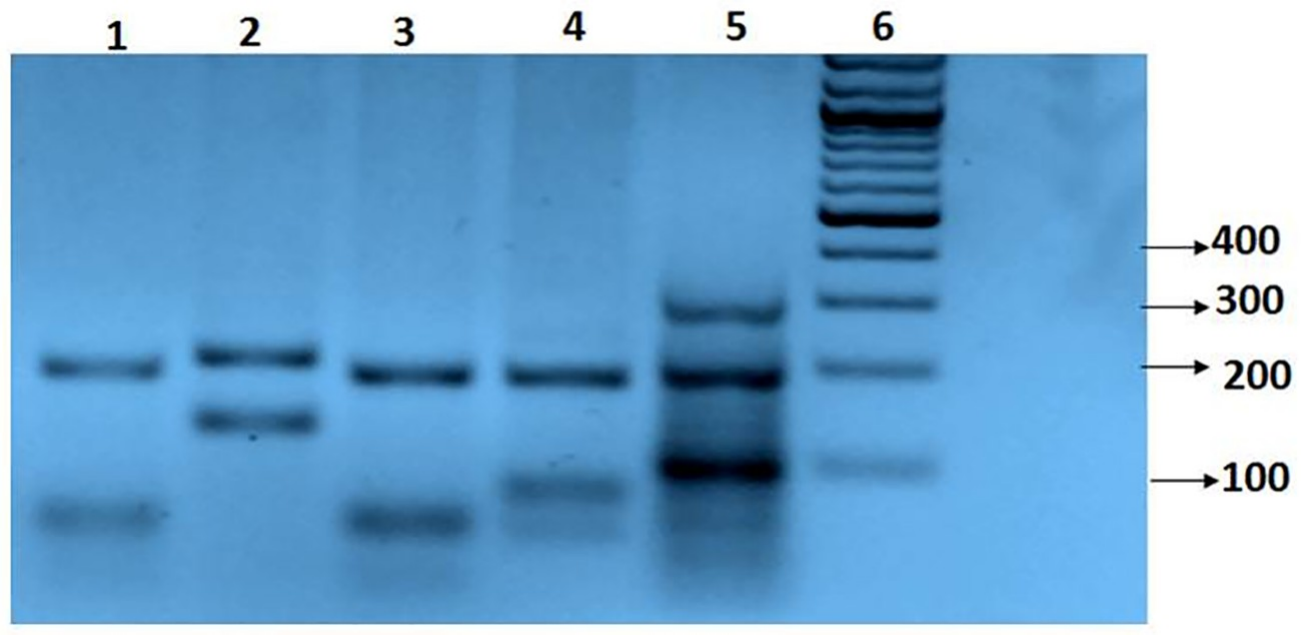
Digestion profile of the ribosomal RNA internal transcribed spacer 1 (ITS1) gene of leishmanial parasites amplified from parasites within sand flies from Palestinian districts, using the restriction enzyme *HaeIII*: Lane 1, a *Ph*. *sergenti* (45A: a representative) sand fly PCR positive for *Leishmania tropica* collected in Jenin District; lane 2, reference strain of *L*. *major* MHOM/SU/1973/5ASKH; lane 3, reference strain of *L*. *tropica* MHOM/1998/LRC-747; lane 4, reference strain of *L*. *infantum* MHOM/TN/1980/IPT1; lanes 5: AQU lab internal marker for 100, 200 and 290bp & 6, 100 bp DNA ladder.

All 691 sand flies were tested individually for the presence of leishmanial DNA, using the ITS1-PCR-RFLP method. Forty-four samples were PCR-positive, giving the expected single band of approximately 330 bp (Fig 4). The digestion of the amplicons with *HaeIII* produced the specific RFLP pattern. Female sand flies can also host other trypanosomatids, which can be amplified by the ITS1 primers [19]. Six of the 29 sand flies collected in the Nablus and Hebron Districts were positive by the ITS1-PCR, but neither the species of their leishmanial infections nor their source of blood meal could be identified. Two other sand flies from Hebron District had fed on human and avian blood.

Only ten leishmanial infections could be identified to the species level in the collected female sand flies, eight of which were identified as *L*. *tropica*. Six *L*. *tropica* infections were detected in female sand flies of the species *Ph*. *sergenti* collected in Jenin District, of which four had fed on human, cow and avian blood. *L*. *tropica* infection was also detected in two female sand flies of the species *Se*. *(Neophlebotomus)* sp. collected in Tubas District, of which one had fed on human blood. Two female sand flies of the species *Ph*. *papatasi* collected in Jenin and Tubas Districts, that had fed only on human blood, had leishmanial infections of *L*. *major* (Table 1).

## Discussion

Previous epidemiological studies undertaken in the Palestinian Authority, especially in the Jenin District, were focused on improving the diagnosis of human cases and the identification of the infective agent. Three species of *Leishmania*: *L*. *major*, *L*. *tropica* and *L*. *infantum*, were identified as causative agents of CL in Palestinian foci [20–25]. Here, data are presented on the distribution of phlebotomine sand flies from areas where sporadic human cases of CL continue to occur every year, giving their rates of infection with leishmanial parasites and their blood feeding preferences.

This study focused on the sand fly species composition and preference of host of the sand fly species present in the Jenin, Tubas, Nablus and Hebron Districts.

Sand flies were collected inside and outside houses, animal shelters and caves. The species *Ph*. *sergenti* and *Ph*. *papatasi* were predominant in collections with sand flies of the species *Ph*. *sergenti* being most abundant near and in rock crevasses inhabited by hyraxes and in the vicinity of houses, and sand flies of the species *Ph*. *papatasi* being most abundant inside houses. Of the 13 sand fly species reported in the Palestinian Authority earlier [26], ten were also identified in this study. The sand fly species *Ph*. *papatasi*, which has already been incriminated in the transmission of the leishmanial species *L*. *major* in the West Bank [27], was clearly the most frequent species. The second predominant species identified in the study area was *Ph*. *sergenti* (21.0%), the confirmed vector of the leishmanial species *L*. *tropica* throughout North Africa, the Middle East and Central Asia [8, 28–30], and also the putative vector of it in the study area [27]. In the Palestinian Authority, especially in the northern part of West Bank, parasites of the species *L*. *tropica* are the most frequent causative agent of human CL: however, the sand fly vectors transmitting them have not been identified with certainty. The species *Ph*. *sergenti* is considered to be the main vector of the species *L*. *tropica* since it is widely distributed and is well adapted to the Mediterranean climate [2, 30].

To detect the presence of leishmanial parasites and identify their species, a kDNA-PCR, which is very good for detecting the parasites in clinical samples because the kinetoplast minicircle DNA is a multicopy target of about 10^4^ minicircles per parasite [31], was combined with an ITS1-PCR-RFLP for species identification, which is more specific but less sensitive. The use of kDNA-PCR is recommended for confirming truly negative samples [21]. This explains the differences seen between the kDNA-PCR and ITS1-PCR infection rates in this study.

Interestingly, DNA from parasites of the species *L*. *tropica* was found in six female sand flies of the species *Ph*. *sergenti*, which corresponds to a rather high infection rate of about 4%. This might be the consequence of high circulation of this species of *Leishmania* in these areas [20, 23, 24].

From 1990–1999, the highest rate of CL was in the vicinity of Jericho (Palestinian Ministry of Health (PMOH)), where the species *L*. *major* was the main cause and where sand flies of the species *Ph*. *papatasi* are abundant, followed by the Jenin District (JD), where the species *L*. *tropica* has been its main cause. Based on records for the years 2002 to 2009, many human cases of CL caused by the species *L*. *tropica* were recorded in the Jenin District with an average annual incidence of 23.0 per 100,000 inhabitants [24]. The species *Leishmania tropica* was identified as the causative agent in 50.7% samples and *L*. *major* in 17.4%. The Palestinian strains of *L*. *tropica* showed more substantial genetic variation than did Israeli strains, which might indicate two different life cycles. Furthermore, analysis of blood meal preferences done here showed that most female sand flies of the species *Ph*. *sergenti* had fed on humans and fewer on hyraxes. Rock hyraxes (*Procavia capensis*) are considered to be the reservoir of the species *L*. *tropica* in the foci in central and northern Israel [2, 32–35]. They live in crevasses among rocks, which afford suitable breeding sites for the sand flies. Svobodova and colleagues [2], found that female sand flies of the species *Ph*. *sergenti* had fed on rock hyraxes more often than on humans [36], which contrasts with our findings. DNA from parasites of the species *L*. *tropica* was not detected in female sand flies of the species *Ph*. *sergenti* and *Se*. (*Neophlebotomus*) sp. that had fed on blood from rock hyraxes here. In 2012, a pilot study was conducted in Zubaidat village north of Jericho and the main focus of the species *L*. *tropica* in the Jordan Valley in order to identify the animal reservoir of the species. However, leishmanial parasites were not detected in nose and ear lesions and internal organs by microscopical examination and using PCR techniques on samples from six rock hyraxes (*Procavia capensis*), five *Rattus* norvegicus and one *Rattus rattus* (Al Jawabreh, personal communication). Here, for the first time, we report the presence of DNA from parasites of the species *L*. *tropica* in a female sand fly of the genus *Sergentomyia* and subgenus *Neophlebotomus* but of unidentified species, where the identification was based on morphological characteristics of spermathecae, cibarium teeth and the length of A3 (Antennal segment 3). Since the detection of Leishmania DNA in an insect never demonstrated this insect could play a role in the Leishmania transmission, this novel finding might give information on its possible role in encouragement the disease transmission in the endemic areas of the Palestinian West Bank.

It is generally accepted that species of *Sergentomyia* do not transmit species of *Leishmania* nor any other pathogens to humans, however, a few recent studies have suggested the possible involvement of some species of the genus *Sergentomyia* in the transmission of leishmanial parasites [37]. In fact, a strain of *L*. *major* was isolated from a female sand fly of the species *Se*. *ingrami* from a Kenyan focus of human CL [38]; and leishmanial promastigotes have been demonstrated in the gut of sandflies of the species *S*. *dubia*, *S*. *schwetzi* and *S*. *magna* in a Senegalese focus of canine leishmaniasis [39]. Also, leishmanial promastigotes were seen microscopically in several species of *Sergentomyia* in Ethiopia [40]. DNA of *L*. *major* was detected in female sand flies of the species *S*. *clydei* in Tunisia, and in female sand flies of the species *S*. *minuta* in Tunisia and Portugal [37, 41]. In addition, DNA from parasites of *L*. *donovani* was detected in female sand flies of the species *S*. *babu* in India [42], Maia and colleagues [43] detected leishmanial DNA phylogenetically related to those species considered pathogenic to humans and dogs in female sand flies of the species *Se*. *minuta* collected in the South of Portugal.

The presence of the species *Ph*. *tobbi* and *Ph*. *perfiliewi*, the presumed vectors of *L*. *infantum* in the Palestinian districts where CL is widespread, underlines the high risk of visceral leishmaniasis occurring in these sites, which are in close proximity to foci of canine visceral leishmaniasis (VL) [24].

To summarize, sand flies of the species *Ph*. *papatasi* were more abundant than those of the species *Ph*. *sergenti* in this focus of CL. Even so, only a few cases were caused by the species *L*. *major* in the Jenin District but this showed that *L*. *tropica* was not the only species responsible for CL there. This has important implications regarding the diagnosis and treatment of leishmaniases acquired in this group of four districts. Observing projects should be advanced to detect the risk of exposure to mixed infections of species of *Leishmania*. For the first time, the presence of parasites of *L*. *tropica* was detected in a sand fly of the subgenus *Se*. (*Neophlebotomus*). This needs further investigation to see if this species is involved in the transmission of CL in the Palestinian West Bank region.

## Conclusions

Application of the RDB blood meal assay proved efficient in analyzing blood meals imbibed by phlebotomine sand flies and those of the genus *Sergetomyia* in identifying the animal hosts from which the blood meals came. Application of the kDNA-PCR and ITS1-PCR-RFLP exposed, in the case of the two PCRs, the presence of leishmanial DNA in some of the blood meals and identification of the species of *Leishmania* by the RFLP analysis. For the first time, the DNA of *L*. *tropica* was detected in a sand fly of the subgenus *Se*. (*Neophlebotomus*) collected in an area where cutaneous leishmaniasis is endemic in the Palestinian West Bank. More research is needed to evaluate its potential role in incubating and transmitting leishmanial parasites. This should include blood meal analysis and parasite isolation on larger numbers of female sand flies of this subgenus. The overall findings of the whole study strongly suggest that human CL caused by *L*. *tropica* is zoonotic in the Palestinian districts that were covered by this study with female sand flies of the species *Ph*. *sergenti* serving as the vector and hyraxes as the reservoir.
